# Towards Targeting the Aryl Hydrocarbon Receptor in Cystic Fibrosis

**DOI:** 10.1155/2018/1601486

**Published:** 2018-02-18

**Authors:** Matteo Puccetti, Giuseppe Paolicelli, Vasileios Oikonomou, Antonella De Luca, Giorgia Renga, Monica Borghi, Marilena Pariano, Claudia Stincardini, Lucia Scaringi, Stefano Giovagnoli, Maurizio Ricci, Luigina Romani, Teresa Zelante

**Affiliations:** ^1^Department of Pharmaceutical Sciences, University of Perugia, 06132 Perugia, Italy; ^2^Department of Experimental Medicine, University of Perugia, 06132 Perugia, Italy

## Abstract

Tryptophan (trp) metabolism is an important regulatory component of gut mucosal homeostasis and the microbiome. Metabolic pathways targeting the trp can lead to a myriad of metabolites, of both host and microbial origins, some of which act as endogenous low-affinity ligands for the aryl hydrocarbon receptor (AhR), a cytosolic, ligand-operated transcription factor that is involved in many biological processes, including development, cellular differentiation and proliferation, xenobiotic metabolism, and the immune response. Low-level activation of AhR by endogenous ligands is beneficial in the maintenance of immune health and intestinal homeostasis. We have defined a functional node whereby certain bacteria species contribute to host/microbial symbiosis and mucosal homeostasis. A microbial trp metabolic pathway leading to the production of indole-3-aldehyde (3-IAld) by lactobacilli provided epithelial protection while inducing antifungal resistance via the AhR/IL-22 axis. In this review, we highlight the role of AhR in inflammatory lung diseases and discuss the possible therapeutic use of AhR ligands in cystic fibrosis.

## 1. Inflammation in Cystic Fibrosis

The inflammatory response is complex and involves a variety of mechanisms to defend against pathogens and repair tissue. In the lung, inflammation is usually caused by pathogens or by exposure to toxins, pollutants, irritants, and allergens. Acute inflammation plays a key role in the innate host defence mechanism yet, when unchecked, may result in tissue destruction and disease. The anti-inflammatory cascade restrains the intensity of the innate response but, when unrestrained, may contribute to chronic inflammation characterized by abnormal wound repair and development of fibrotic disease. So, a delicate balance between inflammation and anti-inflammation is fundamental for lung homeostasis, and understanding cellular and molecular mechanisms behind this balance will enhance diagnosis and treatment of inflammatory and fibrotic lung diseases.

Pulmonary diseases are characterized by the abnormal accumulation and persistence of cells of the acute—such as pneumonia and cystic fibrosis (CF)—or chronic—such as asthma and chronic obstructive pulmonary disease (COPD)—inflammatory response, suggesting that the failure of homeostatic control can contribute to initiation and progression of disease. Inflammation, either secondary to chronic infection or primarily due to cystic fibrosis transmembrane conductance regulator (CFTR) mutations [1], significantly contributes to disease progression, and its control is crucial for improving patient outcomes [2]. Persistent high-intensity inflammation is not only ineffective at clearing pathogens but also leads to permanent structural damage of the airways and impaired lung function. Several defective inflammatory responses have been linked to CFTR deficiency including innate and acquired immunity dysregulation [3]. The inflammation of the CF lung is dominated by neutrophils that release oxidants and proteases, particularly elastase that correlates with lung function deterioration and respiratory exacerbations. Anti-inflammatory therapies are therefore of particular interest for CF lung disease, but abrogating neutrophil and inflammatory response may bear the inherent risk of unleashing bacterial and fungal infections [4]. Thus, despite a plethora of proinflammatory innate immune pathways having been studied and determined as playing a significant role in CF lung disease, therapeutic exploitation of these pathomechanisms remains scarce and therapeutic interventions to dampen inflammation in CF remain an appealing yet challenging approach [4].

## 2. The Aryl Hydrocarbon Receptor

The aryl hydrocarbon receptor (AhR) is highly conserved through evolution [5] and is expressed in the majority of immune cell types and human tissues [6, 7]. AhR is a basic helix-loop-helix (bHLH) transcription factor that has profound effects upon the immunological status of the gastrointestinal and respiratory tracts, establishing and maintaining signaling networks, which facilitate host/microbe homeostasis at the mucosal interface, via regulation of epithelial barrier integrity, of bacterial phyla, and protection from pathogenic insults [6]. Ligand binding to the AhR results in chaperone shedding and translocation to the nucleus where it dimerizes with AhR nuclear translocator (ARNT) and binds to a DNA enhancer sequence, known as dioxin response elements (DREs), within an array of target genes encoding for phase I and phase II metabolizing enzymes (e.g., CYP1A1), as well as the AhR repressor protein (AhRR). The AhRR competes with AhR for binding to ARNT, and this AhRR/ARNT heterodimer binds to DRE and represses transcription. Finally, after ligand-binding nuclear translocation-transcriptional activation, AhR is rapidly degraded [6].

Many genes that participate in immune responses have DRE sequences in their promoters and are responsive to AhR ligands [6]. Thus, AhR is a crucial regulator of the immune system. It is expressed in cells involved in innate and adaptive immunity, such as antigen-presenting cells, mast cells, and ROR*γ*t+ innate lymphoid cells (ILC), the development and function of which is dependent on AhR [8]. Furthermore, the AhR contributes to Th cell differentiation, including FoxP3+ regulatory T cells (Tregs) and IL-10-producing Tr1 cells, Th17 and Th22 cells [9, 10]. Thus, the AhR serves as a sensor that responds to signals, both from the outside environment or internal milieu, to modulate an immune response. This is of evolutionary benefit for individuals exposed to pollution or other toxins, given the ability of AhR to modulate antimicrobial activity via Th17 cell activation, epithelial cell repair, and protection via IL-22 production, and control of inflammation via activation of Tregs. In addition, activation of the AhR causes an upregulation of cytochrome P450 enzymes that metabolize the harmful toxicants. Thus, the AhR has multitasking activities that include antimicrobial defence, tissue protection, repair, and toxin clearance. This creates numerous exciting opportunities to harness the immunomodulatory action of AhR to adapt host responses to infection.

In this regard, numerous naturally occurring endogenous and exogenous AhR ligands have been identified [11–13], with the toxicant 2,3,7,8-tetrachlorodibenzo-*p*-dioxin (TCDD, dioxin being the prototypic AhR ligand (Table 1)). These low molecular weight compounds include indoles, tetrapyrroles, arachidonic acid metabolites, and trp metabolites such as FICZ (6-formylindolo[3,2b]carbazole), ITE (2-(1H-indol-3-ylcarbonyl)-4-thiazol carboxylic acid methyl ester), indirubin, I3C (indole-3-carbinol), and BaP (benzo(a)pyrene) [11, 13–15]. Among these, ITE was isolated from the porcine lung [16]. After binding to the AhR, these ligands promote the activation of various signaling cascades [17, 18]. This may explain why different ligands are able to either ameliorate or conversely aggravate inflammation or autoimmunity [19]. Thus, depending on the nature of ligands, the extracellular and intracellular environments, and the model of pathology considered, the AhR biology is both context- and disease-dependent [10, 20, 21]. This implicates that we must differentiate between the dose- and cell-specific potential triggered by toxic ligands and the physiological effects triggered by endogenous ligands.

## 3. AhR and Lung Inflammation

The recognition of AhR as a master regulator of mucosal barrier function suggests that the respiratory tract is sensitive to AhR signalling and function [22]. The lung is sensitive to AhR ligands, and AhR modulates the immune response in various respiratory diseases [22]. Therefore, AhR ligands (e.g., dietary substances, tryptophan photoproducts, and environmental pollutants) have the potential to be involved both as tools for comprehending the role of the AhR in lung inflammation and as therapeutics for the treatment of various inflammatory lung diseases.

AhR ligands have proven to be beneficial in asthma. TCDD, curcumin (an AhR ligand rich in Indian spice), and quercetin (a plant-derived flavonoid with AhR agonistic activity) all suppress allergic airway inflammation in rodents—reviewed in [22]. The I3C (a dietary compound produced through the breakdown of cruciferous vegetables) attenuated allergic asthma in mice by switching the cytokine response away from Th2 towards a Th1 type of response [22]. However, although Th2 cells are a major source of allergy-promoting cytokines, type 2 ILC and Th17 cells also lead to disease pathology [23–27]. In particular, ROR*γ*t+ ILC and type 2 ILC express AhR [28–30], and levels of both AhR mRNA and IL-22 protein are markedly boosted in asthmatic patients [22]. However, the functions of the AhR in lung ILCs is still poorly defined. Similarly, albeit TCDD and other AhR ligands ameliorated allergic inflammation in murine lung diseases, details about the effects on pulmonary Th cell differentiation are still to be clarified [22]. Taken together, AhR ligands may play an important role as modulators of immunological responses that contribute to allergic events. However, differences in metabolism, AhR binding, and downstream gene activation indicate the need to better analyze the role played by AhR in allergic asthma.

Given that COPD has been associated with chronic exposure to lung irritants from cigarettes, biomass burning, air pollution, and dust [31], an immunomodulatory activity of the AhR is plausible. Not surprisingly, AhR signaling both promoted and attenuated the expression of inflammatory cytokines and matrix metalloproteases in murine lung by acting on innate immune cells variably involved in COPD, such as macrophages, neutrophils, mast cells, epithelial cells, and fibroblasts—reviewed in [22]. For instance, mast cells produce IL-17 in response to AhR stimulation and AhR/IL-17 double-positive mast cells are increased in the bronchial lamina propria of COPD patients [32]. In murine cigarette smoke-induced COPD, the COPD pathogenesis was markedly enhanced in the absence of AhR via mitochondrial dysfunction, decreased levels of antioxidant regulating proteins (i.e., MnSOD and CuZn-SOD) [33], and increased Th1, Th2, and Th17 cells [22]. Recently, smoking was found to be associated with relevant alterations in methylation, especially at the AhRR [34]. These evidences suggest that changes in AhR expression and function may be a risk factor for COPD and other lung inflammatory diseases in smokers and that the AhR status in smokers could be dysregulated. Of interest, the activity of the indoleamine 2,3-dioxygenase (IDO)1 enzyme and the IL-10/IL-17 ratio were both decreased in COPD patients [35], a finding further pointing to AhR, known to promote IDO1 activity [36], as a master regulator of inflammation and tolerance in the lung.

The ability of the AhR pathway to crosstalk with other pathways such as hypoxia signaling pathway [37] would suggest a biological function in CF in which hypoxemia has been described at the tissue and cellular levels [38]. Low tissue oxygen levels induce expression of hypoxia-inducible factor (HIF), a nucleoprotein consisting of the oxygen-regulated HIF-1*α* and the constitutively expressed ARNT or HIF-1*β* [39]. HIF-1*α* is continuously synthesized but under normoxic conditions is targeted for ubiquitination and proteasomal degradation. In contrast, under hypoxic conditions, HIF-1*α* is stabilized, dimerizes with HIF-1*β*, binds coactivators of hypoxia response elements, and regulates the transcription of hypoxia-regulated genes that mediates adaptive responses to ensure cellular survival under hypoxic conditions but also dysregulated inflammation. Indeed, the increased HIF-1*α* in CF correlated with increased activation of the inflammatory receptor of advanced glycation end products (RAGE), an important mediator of airway inflammation in CF and other lung diseases [38, 40]. The important role of ARNT in both the AhR and HIF-1*α* signaling pathways establishes a meaningful foundation for a possible crosstalk between these two vitally important signaling pathways. This crosstalk might lead to interference between the two signaling pathways and thus might play a role in the variety of cellular responses after exposure to AhR ligands and reduced oxygen availability.

Collectively, these studies highlight the potential differences that can occur in the lung following AhR activation by disparate agonists and further emphasize the need for extensive evaluation of the different AhR ligands.

## 4. Microbial-Derived Indoles as Therapeutic AhR Ligands

Ligands required to activate intestinal AhR derive from ingestion of plant-derived dietary ligands such as polyphenolic flavonoids (e.g., quercetin) or glucobrassicin-derived gastric acid condensation products (e.g., indolo-[3,2b]carbazole) or endogenously produced. For example, kynurenic acid and kyneurenine, products of tryptophan IDO1 and tryptophan pyrolase metabolic pathways, have been established as AhR agonists [11, 15]. Additional examples of microbial AhR agonist production occur at other barrier tissues, such as the skin where indirubin and malassezin produced by the yeast *Malassezia* are both potent AhR activators [41] and the lung where AhR sensing of the bacterial pigments phenazines regulates antibacterial defence [42].

**Figure 1:**
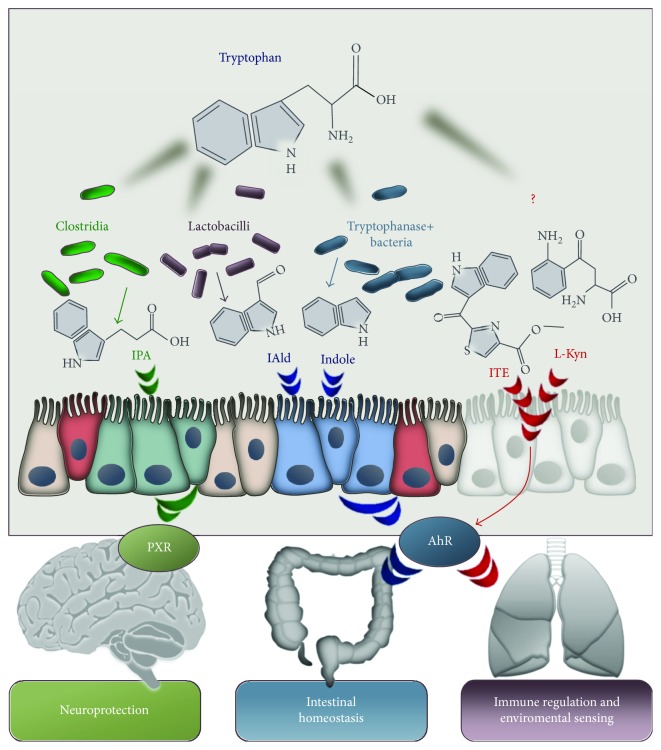
Tryptophan degradation pathways. The image shows how different microbes are involved in several pathways of tryptophan degradation leading to the production of immunoreactive metabolites acting locally and systemically as agonists of PXR and AhR. IPA: indole-3-pyruvic acid; IAld: indole-3-aldheyde; ITE: 2-(1H-indol-3-ylcarbonyl)-4-thiazol carboxylic acid methyl ester; L-Kyn: L-Kynurenine; PXR: pregnane X receptor; AhR: aryl hydrocarbon receptor.

As already mentioned, a number of studies have highlighted the capacity of AhR to respond to indolyl metabolites, including indoxyl-3-sulfate, 6-formylindolo[3,2b]carbazole, kyneurenine, kynurenic acid, tryptamine, indole-3-acetate, and dietary indoles (indole-3-carbinol and 3,3′-diindolylmethane from the Cruciferous vegetables), thus positioning the AhR as a candidate indole receptor [11, 15, 43, 44]. The aromatic bicyclic indole—made of benzene fused to a pyrrole ring—is abundantly present in nature as a metabolic derivative and as an indolyl moiety present in biological molecules utilized by plants, animals, and microbes. Functionally, indole characterizes the essential amino acid trp and is an important chemical constituent of the hormone melatonin and of auxin and indigo that are a plant signaling molecule and a natural pigment, respectively. Soils and the gastrointestinal tracts of animals contain high levels of indole, and it is now evident that indole and its derivatives are considerably more important as interkingdom signals than was originally believed. As an intercellular and interspecies bacterial signaling molecule, indole plays many roles in bacterial pathogenesis [45, 46]. Bacterial indole and indolyl compounds behave as quorum-sensing molecules among bacterial populations and are implicated in motility, virulence, biofilm formation, and antibiotic resistance [45, 46]. The gastrointestinal tract harbors numerous species (e.g., *E*. *coli*) which enable to synthesize indole through the tryptophanase- (TnaA-) dependent metabolism of tryptophan [47], so high micromolar concentrations of indole are detected in the gut lumen and feces reviewed in [45]. New findings have highlighted that microbial-derived indoles also mediate signaling between intestinal microbiota and the host via AhR, leading to mucosal protection and regulation of local and distant inflammation [48] (Figure 1).

**Figure 2:**
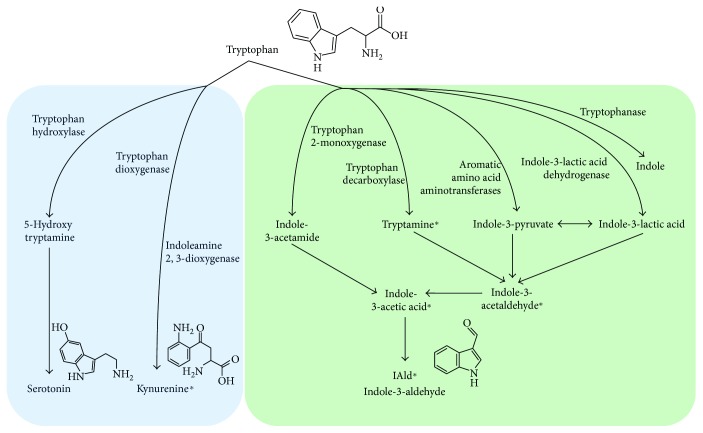
Tryptophan metabolic pathways. The image shows enzymes involved in the host's pathways (on the left) and microbial pathways (on the right) of tryptophan metabolism. Asterisks indicate metabolites with AhR agonistic activity.

The AhR ligand ITE, found in the mammalian lung [16] exhibited important immunomodulatory properties both locally and at distant sites [49], apparently by enhancing Treg activity and reducing Th17 cell function. Therefore, AhR ligands are promising therapeutic compounds for lung diseases. However, more research is required to understand the multifaceted role of AhR in the context of inflammatory lung diseases and to define AhR ligands with safe pharmacological profiles in clinical practice. By adopting a “top-down strategy” to screen host/biofluids/tissues for component of microbial origin, a microbial tryptophan metabolic pathway leading to the production of indole-3-aldehyde (3-IAld, Figure 2) has recently been identified that preserves immune physiology at mucosal surfaces while inducing anticandidal resistance via AhR [50]. Much like probiotics, 3-IAld fulfilled the requirement of protecting and maintaining mucosal integrity during fungal infections or chemical damage [51]. Our preliminary results suggest that 3-IAld may protect from respiratory allergy in murine CF and exerts potent antimicrobial activity against gram+ and gram− bacteria (data not shown). Thus, the 3-IAld/AhR axis could be therapeutically exploited for tissue immune homeostasis, microbial symbiosis, and pathogen resistance in CF and other respiratory diseases.

## 5. Conclusion

It is clear that the AhR and its ligands have important immunomodulatory properties that fine-tune the respiratory immune response. A deeper knowledge of AhR ligands and AhR-signaling properties relative to the development of therapeutic and preventive approaches to inflammation and fibrosis is required. In this regard, the well-known anti-inflammatory activity of lipoxin, a known AhR ligand [52], in CF [53] points to the drugability of the AhR pathway. In addition, considering the epigenetic regulation of the CFTR transcription [54], this suggests that the therapeutic targeting of AhR will encompass epigenetic mechanisms of CFTR regulation. We are only beginning to understand the effects of indole and indole-producing bacteria on human health. Despite the efforts made to elucidate the mechanisms of action of indoles, the genetic and molecular mechanisms of indole signaling remain unclear. Hence, more research is required to understand the multifaceted role of the AhR in the context of inflammatory lung diseases, as well as the specificity, pharmacology, and dose effect of indoles in the different inflammatory lung diseases. It is clear that an efficacious AhR ligand may not exhibit a “one-size-fits-all” role for limiting inflammation.

## Figures and Tables

**Table 1:** List of major ligands of aryl hydrocarbon receptor.

Source	Activity	Examples
Agonists		
Xenobiotics	Strong	*Halogenated aromatic hydrocarbons*
		2,3,7,8-Tetrachlorodibenzo-*p*-dioxin
		Dibenzofurans
		Biphenyls
		*Polyaromatic hydrocarbons*
		3-Methylchlolanthrene
		Benzo(a)pyrene
		Benzanthracenes
		Benzoflavones
		*Pharmaceuticals*
		Tranilast
		Leflunomide
		Omeprazole
Dietary	Weak	*Flavonoids*
		Quercetin
		Indole-3-carbinol
		3,3′-Diindolylmethane
		Indolo[3,2b]carbazole
Endogenous	Weak	*Tryptophan metabolites*
		Kynurenic acid
		Kynurenine
		Tryptamine
		6-Formylindolo[3,2b]carbazole
		Indoxyl sulfate
		*Microbiota*
		3-Methylindole
		Tryptanthrin
		1,4-Dihydroxy-2-naphtoic acid
		Indole-3-aldehyde
		Indole-3-acetate
		Phenazines
		Indirubin
		Malassezin

Antagonists		
Xenobiotic	Weak	3,4-Dimethoxy-a-naphthoflavone
		6-Methoxy-1,3,8-trichlorodibenzofuran
	Strong	CH-223191
		StemRegenin 1
Dietary	Weak	Resveratrol

SAHRM (Agonists/Antagonists)		
Xenobiotic	Weak	3,4-Dimethoxy-a-naphthoflavone
		6-Methoxy-1,3,8-trichlorodibenzofuran
Endogenous/Dietary	Weak	Indole
		Quercetin
		Apigenin

SAHRM: selective AhR modulator.
